# Spatial Distribution and Temporal Dynamics of Neomycin-Induced Neuromast Cell Damage and Regeneration in the Mexican tetra (*Astyanax mexicanus*)

**DOI:** 10.3390/cells14211680

**Published:** 2025-10-27

**Authors:** Gandhrav Goel, Nitesh Sanghai, Geoffrey K. Tranmer, Devi Atukorallaya

**Affiliations:** 1 Department of Oral Biology, Dr. Gerald Niznick College of Dentistry, Rady Faculty of Health Sciences, University of Manitoba, Winnipeg, MB R3E 0W2, Canada; 2 College of Pharmacy, Rady Faculty of Health Science, University of Manitoba, Winnipeg, MB R3E 0T5, Canada

**Keywords:** neomycin, Mexican tetra fish, neuromast cells, lateral lines, inner hair cell regeneration

## Abstract

**Highlights:**

- Neuromast cells in the Astyanax mexicanus lateral line system are sensitive to neomycin (Aminoglycosides).
- Neuromast cells disappeared within 24 h of neomycin treatment and returned to baseline levels by 72 h post-exposure.
- Gene expression levels of fgf1 and axin2 showed a slight increase during neuromast cell regeneration following neomycin exposure, whereas sox2 expression remained relatively unchanged.

**Abstract:**

Neuromast cells are specialized mechanosensory receptor cells embedded within the lateral line system of aquatic vertebrates, enabling the detection of water movement and vibration that are essential for navigation, prey capture, and predator avoidance. These cells share common evolutionary and functional homology with mammalian inner ear hair cells, both of which rely on stereocilia-mediated mechano-transduction and ion channel activation to convert mechanical stimuli into neural signals. Unlike their mammalian counterparts, neuromast hair cells possess a regenerative capacity following damage, making the lateral line system a unique model for studying hair cell regeneration and sensory restoration. This study examines the potential of the Mexican tetra (*Astyanax mexicanus*) as a novel model organism for investigating ototoxicity and regeneration of neurosensory hair cells. Here, we explore the cranial and trunk lateral line neuromasts, including deep canal neuromast cells located in facial bones, such as the mandible and circumorbital bones. In the present study, juvenile surface-dwelling Mexican tetra were exposed to a 500 µM neomycin for 4 h to induce targeted hair cell damage. The samples were collected at 4-, 12-, 24-, and 72 h post-exposure. Furthermore, neuromast cell viability was assessed using [2-(4-(Dimethylamino) styryl)-N-ethylpyridinium iodide] (DASPEI). Gene expression analysis revealed a modest increase in Fibroblast Growth Factor 1 (*fgf1*) and Axis Inhibition Protein 2 (*axin2*) expression following treatment; however, these changes were not statistically significant. The SRY-box transcription factor 2 (*sox2*) remains constant throughout the exposure and recovery period. These findings highlighted the regenerative dynamics of neuromast cells in Mexican tetra. This work lays the foundation for future therapeutic strategies targeting human sensory deficits, particularly those involving inner ear hair cell degeneration.

## 1. Introduction

Neuromast cells are types of neurosensory hair cells and can be found within the lateral lines system of aquatic vertebrates [1,2]. Two groups of neuromasts are generally classified according to their location. (1) Canal neuromasts, which are enclosed in canals, and (2) smaller superficial neuromasts, which are found within the skin epithelium. In juvenile and adult bony fishes, canal neuromasts are found in the epithelium lining of the lateral line canals, which are integrated into a subset of dermal bones of the skull (the cranial lateral line canals) and in the lateral line scales on the trunk (trunk canal). Superficial neuromasts are found on the skin surface, and may be associated with lateral line canals.

Morphologically, a neuromast unit is composed of a cluster of hair cells surrounded by supporting cells, all embedded within a gelatinous structure called the cupula. The hair cells are responsible for detecting water movement and pressure changes in the surrounding environment. Each hair cell features a bundle of stereocilia, actin-rich projections arranged in a staircase-like pattern, ranging from approximately 1 to 10 μm in length. Adjacent to the tallest stereocilia is a single kinocilium, typically 10 to 15 μm long, which serves as a directional reference point. These hair bundles are embedded in a gelatinous structure called the cupula, which transmits mechanical stimuli caused by water flow. When the cupula is displaced, the stereocilia bend toward or away from the kinocilium, triggering ion channels to open or close and initiating electrical signals that are sent to the brain. At least two types of support cells can be identified: the first type, called support cells, are localized at the base of the neuromast and are interposed to the hair cells; the second type, the mantle cells, are placed around the support cells and laterally complete the mechanosensory organ. These cells provide structural integrity and provide new cells during hair cell regeneration [1,3]. Canal neuromast cells share significant structural and functional similarities with hair cells in the human inner ear cells, reflecting a conserved mechanosensory organogenesis across vertebrates [1,3,4,5,6].

Neuromast cells are found to be robust, with increased plasticity, in which exact numbers cannot be determined through age and size of the fish [6]. The early development of neuromast cells occurs through the migration of the primordium (a cluster of precursor cells) along the posterior lateral line. As the primordium moves in an anterior-to-posterior direction, it deposits clusters of cells at regular intervals, which subsequently differentiate into neuromast cells [1,2,3,4,6].

Neuromast cells are found to be robust, with increased plasticity, in which exact numbers cannot be determined through age and size of the fish [7]. The early development of neuromast cells occurs through the migration of the primordium (a cluster of precursor cells) along the posterior lateral line. As the primordium moves in an anterior-to-posterior direction, it deposits clusters of cells at regular intervals, which subsequently differentiate into neuromast cells [1,2,3,4,7].

Recently, Mexican tetra (*Astyanax mexicanus*) have been identified as a novel model organism in developmental biology. This species exists as two morphs: surface fish and cave fish. Both of which come from the same species but evolve differently due to physical isolation within barriers, giving them different phenotypic traits. The surface fish that can be found in the freshwater streams of Mexico have body pigmentation and eyes. The cave fish have no eyes and pigments [8,9]. Cave fish exhibit morphological and sensory adaptations suited to their nutrient-scarce environments, such as enlarged jaws and an increased number of teeth and taste buds, which enhance their ability to scavenge and extract nutrients from limited food sources [9,10]. Little work has been performed to explore the use of the Mexican tetra as a model organism for epithelial organ regeneration or the sensory hair cell studies. The anatomical location and morphology of epithelial appendages, such as teeth, taste buds, and neuromast cells, differ significantly between the two morphs, reflecting their distinct evolutionary trajectories. These variations are a direct consequence of adaptive divergence, with each morph developing specialized traits suited to its unique environmental pressures [8,9,11]. Cave fish exhibit an increase in both the size of the olfactory lobe and the number of neuromast cells compared to surface fish, resulting in heightened sensitivity to vibration-related stimuli and enhanced environmental perception [8,12]. Understanding the evolutionary adaptations between the two subspecies, along with identifying conserved molecular mechanisms, may offer valuable insights that contribute to the development of future medical therapies. The existing literature has shown that similar chemicals that damage mammalian hair cells in humans can also cause neuromast degeneration in lower vertebrates. In a study by Fan et al., larval Siberian sturgeon (*Acipenser baerii*) exposed to neomycin exhibited significant hair cell loss in both mechanoreceptor (neuromast) and electroreceptor (ampullary) organs, with the lowest cell counts observed at 6 h post-treatment and complete regeneration achieved within 7 days [13]. Complementing these findings, Montalbano et al., investigated zebrafish lateral line hair cells and revealed drug-specific and topographical sensitivity to neomycin and gentamicin [14]. Their results showed that neomycin caused more pronounced hair cell death than gentamicin at equal concentrations, and that L1 neuromasts (on the trunk) were more susceptible than T1 neuromasts (on the tail) [14], particularly under gentamicin exposure. These studies highlight use of these models for research in the fields of ototoxicity and regenerative studies [4,5,15,16,17,18]. In this study, we utilize the surface-dwelling Mexican tetra (*Astyanax mexicanus*) to investigate ototoxicity and neurosensory hair cell regeneration, leveraging its established use in our laboratory and its anatomical and physiological comparability to zebrafish. The surface-dwelling Mexican tetra (*Astyanax mexicanus*) is better suited for quantitative analysis of cranial neuromast cells compared to its cave-dwelling counterpart due to its more consistent craniofacial morphology. While cave fish possess a higher number of cranial neuromasts, their extensive variation in facial bone structure and neuromast distribution introduces significant variability that complicates standardized measurements. In contrast, surface fish exhibit stable and symmetrical cranial anatomy, allowing for more reliable quantification and reproducibility in neuromast-based assays. By positioning A. mexicanus alongside zebrafish in comparative analyses, we aim to expand the repertoire of model organisms available for ototoxicity research and uncover conserved and divergent mechanisms of hair cell resilience and recovery.

## 2. Materials and Methods

### 2.1. Mexican tetra Fish Rearing and Breeding

Mexican tetra (*Astyanax mexicanus*) surface fish were housed on a 12 h day/night cycle at the temperature of 21 °C in the Tecniplast rack system at the University of Manitoba. The water conductivity of the system was set to 900 µS, and the pH was set to 7.2. Adult Mexican tetra fish {more than 6 months post-fertilization (mpf)} were fed twice daily with flake food (Tetramin). To set up a breeding tank, the water temperature was set to 26 °C, and male and female fish were placed in a ratio of 2:1, respectively. Breeding was expected to occur within the following three days, during which laid eggs were carefully monitored and collected for the experiment. The eggs were incubated at a temperature of 26 °C. Larval fish were fed with live brine shrimps (*Artemia fransicana*) and gradually transferred to larger containers at 30 dpf. Juvenile fish (1 mpf to 6 mpf), with the average standard length of 3 cm, were raised in the regular system tank. All animal research was performed according to standard conditions and guidelines of the Canadian Council of Animal Care (CCAC). All protocols were reviewed and approved annually by the University of Manitoba Central Animal Care Services.

### 2.2. Neomycin Treatment

Neomycin is a broad-spectrum aminoglycoside antibiotic known to be ototoxic in humans. Neomycin Sulfate 500 μM concentration (Cas-No. 1406-10-3 Millipore Sigma, Saint Louis, MO, USA) exposure was conducted on 30 dpf surface fish (*n* = 20) for 4 h. The samples were washed three times thoroughly after the exposure time period and used for further analysis.

### 2.3. DASPEI (2-[4-(dimethylamino)styryl]-N-ethylpyridinium iodide) Staining and Imaging

DASPEI (Cas: 3785-01-1 Millipore Sigma) is a neuromast-specific live staining. A DASPEI solution was prepared at a concentration of 50 µg/mL. Each individual sample was exposed for 1 h with observations recorded at 4-, 12-, 24-, and 72 h post-exposure. Immediately after the treatment of DASPEI, post-staining samples were sacrificed using 0.1% MS222 (Tricaine Methanesulfonate). Imaging was performed to locate and quantify the neuromast cell presence of each individual sample on the circumorbital areas and the lateral side of the body surface. The samples were photographed using Leica stereo microscope with Texas Red wavelength (596 nm). Normalization of neuromast cells was performed through counting cells within a specific cranial region specifically on infraorbital bone3. The trunk neuromast cells were counted between the second and third canal neuromast cell areas.

Neuromast cell visualization and counting was further performed on DASPEI and Alizarin Red live staining. The fish were immersed in 1 h in Alizarin Red followed by DASPEI stain. The samples were then euthanized and visualized under the stereomicroscope and the counting was performed manually. The confocal image was used to further quantify the number of the superficial and canal neuromast cells. (Supplementary Figures S1 and S2).

### 2.4. Primer Design

Genes were selected from the NIH GenBank (Table 1). *fgf1a* (XM_007258578.4), *axin2* (XR_007427266.1) and *sox2* (NM_001319965.1). Primer-BLAST (version 2.5.0) tool was utilized to generate primers of 70–200 bp in length, T_m_ ranging for 57.0–63.0 °C, while excluding other genes from the *Astyanax mexicanus*, avoiding non-specific binding, with previously established GAPDH found within the lab. Integrated DNA TECHNOLOGIES primer analysis tool was used to account for primer dimers as well as hairpin formation before ordering.

### 2.5. RNA Isolation/QPCR Analysis

RNA isolation was performed at 24 h post-treatment and 72 h post-treatment compared to the control. Samples {control (n = 4) and post-treatment (n = 3)} were sacrificed and were transferred and stored in RNAlater (Ref # AM7020 Invitrogen Thermo Fisher Scientific, Waltham, MA, USA). Samples were dissected to isolate the left and right cranial regions, encompassing the facial epithelium and underlying dermal bones. These regions included both superficial neuromast cells embedded within the skin and canal neuromasts housed in the bony lateral line canals. Qiagen RNeasy Mini Kit (Venlo, The Netherlands) (50) was used for RNA extraction. Individual samples were measured through Nano-drop to ensure appropriate RNA was available for cDNA synthesis (1500 ng) and qPCR. cDNA synthesis was performed using iScript™ cDNA Synthesis Kit (Cat #: 1708891 BIO-RAD, Hercules, CA, USA)). Real-time PCR was conducted using iScript™ Reverse Transcription Supermix for RT-qPCR (Cat #: 1708841 BIO-RAD, Hercules, CA, USA) and QuantStudio™ 5 Real-Time PCR System, 96-well, 0.1 mL by Thermo Fisher Scientific Waltham, MA, USA was utilized to perform real-time PCR, data for which was normalized using GAPDH.

### 2.6. Phylogenetic Analysis

Genes of interest were identified using GenBank, specifically *fgf1*, and BLAST was employed to locate related genes within established model organisms. Percent similarity was considered as well as the length of the full transcript, in case multiple variants were found. When considering different variants with species and transcripts of the same gene, percent similarity was considered. IQtree was utilized using the maximum likelihood model with 10,000 bootstrapping to obtain a phylogenetic tree. This was visualized using Figtree.

### 2.7. Statistical Analysis

Analysis was performed through graph pad using the one-way ANOVA to determine *p*-values within each time point. For the qPCR examination, the genes of interest were standardized against GAPDH as the housekeeping gene and calculated for ΔCq and 2^ΔCq^ to examine changes in relative expression.

## 3. Results

### 3.1. Phylogenetic Analysis Data

**Figure 1:**
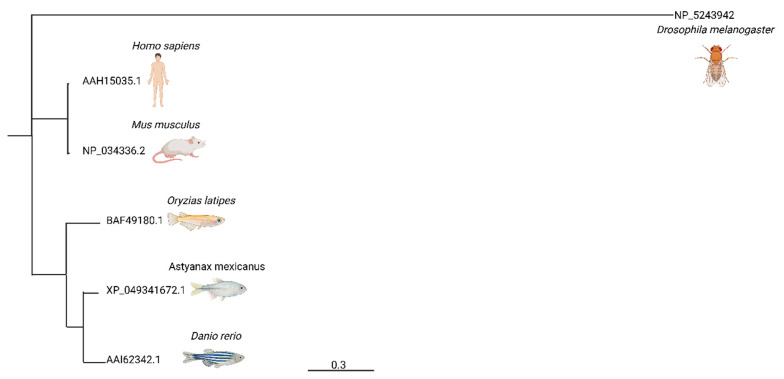
Phylogenetic tree of the Fibroblast Growth Factor 1 (*fgf1*). The phylogenetic tree was constructed to assess the percent similarity of the *fgf1* gene among commonly used model organisms in ototoxicity research. Here we have compared human, *Astyanax mexicanus*, *Drosophila melanogaster*, *Mus musculus*, *Oryzias latipes*, and *Danio rerio*.

The distance between each individual organism through the length of tree branch, represented by genetic exchange of *fgf1*. *Drosophila melanogaster* is the furthest from humans that is widely used for ototoxic studies Figure 1. *Mus musculus* being the closest, then *Oryzias latipes*, following the *Astyanax mexicanus* and *Danio rerio*. *Danio rerio* and *Astyanax mexicanus* are relatively close neighbors, with no notable difference being present within the two lower vertebrates.

### 3.2. Effect of Neomycin Exposure on Neuromast Cells Within Infraorbital Bone3

Mexican tetra surface fish (n = 5) were isolated and exposed to 500 μM neomycin for 4 h. Immediately after treatment, surface fish were live stained with 50 μg/mL DASPEI for one hour at individual time points of 4 h, 12 h, 24 h, and 72 h post-neomycin-treatment. Subsequently, fish were imaged via stereomicroscopy, and neuromast cells located on infraorbital bone3 were visualized and quantified using ZEN 3.4 software under 596 nm red light illumination.

**Figure 2:**
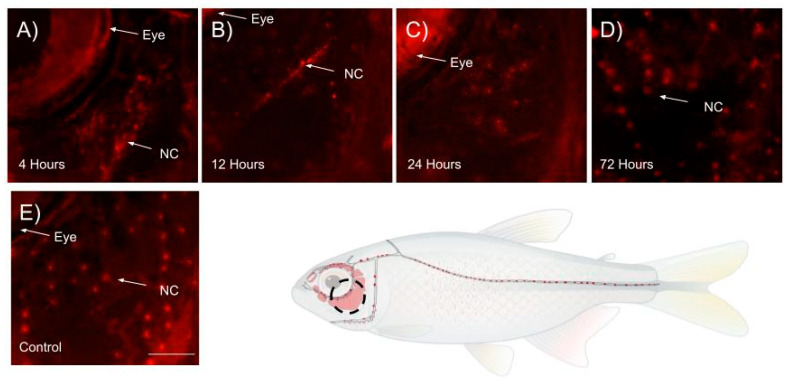
Change in neuromast cells within infraorbital bone3 regions of surface fish treated with 500 µM neomycin. Neuromast cells (NC) are signified by the white arrow pointing towards the red dot-shaped structures upon the infraorbital bone3. (**A**) 4 h post-neomycin treatment, (**B**) 12 h post-neomycin treatment, (**C**) 24 h post-neomycin treatment, (**D**) 72 h post-neomycin treatment, and (**E**) control sample without neomycin treatment. Scale bar 500 µM.

**Figure 3:**
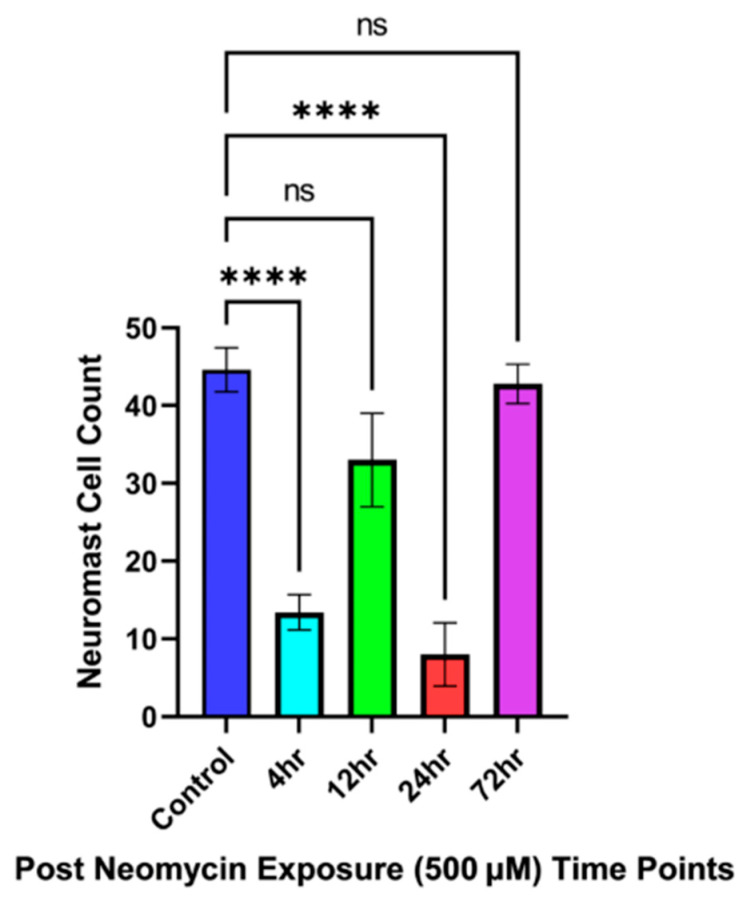
Quantification of infraorbital bone3 neuromast cells following neomycin exposure at multiple time points. The data represents the neuromast cell after neomycin post-exposure relative to the control. The data were analyzed using one-way analysis of variance (ANOVA) followed by Dunnett’s multiple comparison tests, with a significance level set at *p* < 0.05. Data is presented as a mean value ± standard error of mean (error bars); where n = 5 data points for each time point. Here, *p* ≤ 0.0001 with **** *p* ˂ 0.0001 versus control and ns—non-significant. Data are representative of five independent experiments. All statistical analyses were performed using GraphPad Prism 8 software (GraphPad Software, San Diego, CA, USA).

Infraorbital bone3 was taken, due to high neuromast cell concentrations being present, as the reference point in the circumorbital region of the fish. A decline in the neuromast cells over the course of 12 h post-neomycin-treatment, with minimal neuromast cell count being seen 24 h post-exposure in Figure 2A, Figure 2B, Figure 2C, and Figure 2D, respectively. Approximately 38–39 neuromast cells are stained with DAPSEI, as in Figure 2E. Figure 3 shows the statistical significance between the control and the time points 12- and 24 h post-treatment. Figure 2D and Figure 3 show no countable neuromast cells 24 h after exposure. By 72 h post-treatment, the neuromast cell count had returned to a relatively normal level, with no significance when compared to the control.

### 3.3. Effect of Neomycin Exposure on Trunk Neuromast Cells

Mexican tetra surface fish were isolated and exposed to 500 μM neomycin for 4 h. Immediately after treatment, surface fish were live stained with 50 μg/mL DASPEI for one hour at individual time points of 4 h, 12 h, 24 h, and 72 h post-neomycin-treatment. Upon live staining, fish were imaged through stereomicroscopy and neuromast cells on the trunk within the lateral lines and superficial surface were visualized and counted using ZEN 3.4 under red light 596 nm.

**Figure 4:**
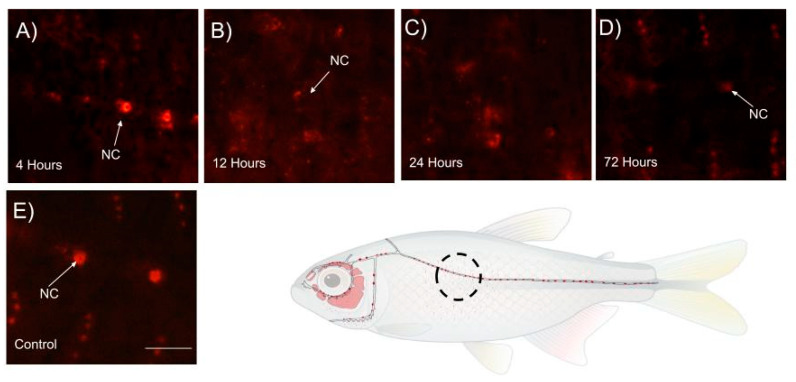
Change in the trunk neuromast cells surface fish treated with 500 µM neomycin. The surface fish were treated with neomycin for 4 h and stained using DASPEI live staining for 1 h. Neuromast cells (NC) are signified by the white arrow in a cubic area of 830 µM × 1220 µM within the body (**A**) 4 h post-neomycin-treatment, (**B**) 12 h post-neomycin-treatment, (**C**) 24 h post-neomycin-treatment, and (**D**) 72 h post-neomycin-treatment. (**E**) Control sample without neomycin treatment, all of which are scaled at 500 µM.

**Figure 5:**
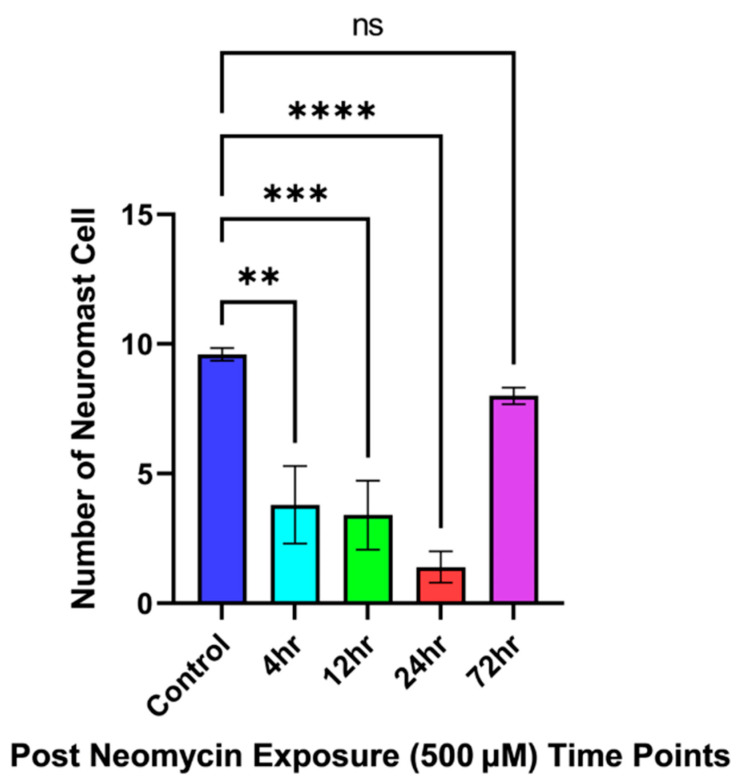
Quantification of trunk neuromast cells within lateral lines following 500 μM neomycin exposure. The data represents the neuromast cell after neomycin post-exposure relative to control. The data were analyzed using one-way analysis of variance (ANOVA) followed by Dunnett’s multiple comparison tests, with a significance level set at *p* < 0.05. Data are presented as a mean value ± standard error of mean (error bars), where n = 5 data points for each time point. Here, *p* ≤ 0.0001, *p* = 0.0006 and *p* = 0.0012 with **** *p* ˂ 0.0001, *** *p* ˂ 0.001, and ** *p* ˂ 0.01 versus control and ns—non-significant. Data are representative of five independent experiments. All statistical analyses were performed using GraphPad Prism 8 software (GraphPad Software, San Diego, CA, USA).

An average of 9–10 cells were visualized through stereomicroscopy within the noted areas seen in Figure 4E and Figure 5. The quantity decreases over 24 h, eventually becoming almost undetectable, with minimal to no cellular staining observed by the final time points shown in Figure 4B–D. Statistically significant differences are observed between the control and 4-, 12-, and 24 h time points after neomycin treatment, as indicated in Figure 5. Similar results are seen when comparing 72 h to 4-, 12- and 24 h post-treatment, with no significant difference between the control and the 72 h time point Figure 5. Overall, these results demonstrate the toxic effects of neomycin on Mexican tetra neuromast cells and their regenerative capacity over 72 h.

### 3.4. Gene Expression Analysis

**Figure 6:**
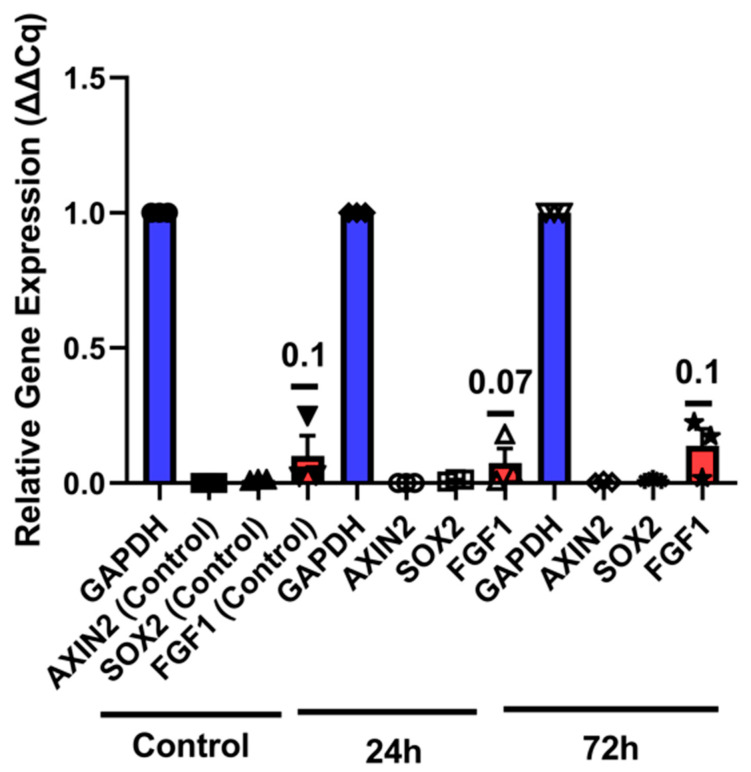
Relative gene expression of Axis Inhibition Protein 2 (*axin2*), SRY-box transcription factor 2 (*sox2*), and Fibroblast Growth Factor 1 (*fgf1*), in control, 24- and 72 h post-neomycin-treatment.

Delta cycle quantification (ΔCq) values are standardized through GAPDH, and relative expression is calculated by 2^ΔCq^ for delta delta cycle quantification (ΔΔCq) to signify relative gene expression; higher values indicate increased expression (Figure 6). Relative gene expression analysis revealed time-dependent increases in *fgf1* {relative mean expression (ΔΔCq) at 24 h (0.07) and 72 h (0.1)} following neomycin treatment. However, one-way ANOVA followed by Tukey’s post hoc test did not reveal statistically significant differences among groups for any of the genes analyzed (*p* > 0.05). These findings suggest potential transcriptional activation of *axin2* and *fgf1* in response to neomycin, though further studies with larger sample sizes are needed to confirm significance.

## 4. Discussion

The present study evaluates the regenerative capacity of neuromast cells in *Astyanax mexicanus* following ototoxic damage. We compared FGF1 over the phylogeny and found that it is essential for neuromast cell development and regeneration. The analysis highlights a close evolutionary relationship between zebrafish and surface-dwelling Mexican tetra, suggesting strong potential for using the Mexican tetra as a model organism in neurosensory hair cell research (Figure 1). This claim is further supported by the significant evolutionary distance between humans and *Drosophila melanogaster*, a widely used model in regenerative science despite its limited genetic proximity. By examining these evolutionary relationships, our study offers new insights into neuromast cell development and regeneration from an evolutionary perspective.

The Mexican tetra stands out as a promising model due to its high fecundity, ease of genetic manipulation, and the non-invasive ability to visualize neuromast cells directly on the surface of the organism. These features make it an ideal candidate for exploring novel mechanisms of hair cell regeneration, particularly in the context of ototoxicity and sensory cell damage. Identification and quantification of sensory hair cell survival in experimental models of ototoxicity relies on fluorescent markers that preferentially label live hair cells. DASPEI dye has been identified as a cationic, lipophilic dye that selectively accumulates in hair cells of the zebrafish lateral line, electroreceptors, and chloride cells [19,20]. The uptake of which is dependent on an intact mitochondrial membrane potential, since its accumulation is driven by the electrochemical gradient across the mitochondrial inner membrane [20,21]. Consequently, the loss of DASPEI fluorescence is interpreted as a proxy for mitochondrial dysfunction and hair cell death, particularly following aminoglycoside drug exposure [21].

However, DASPEI labeling is selective for hair cells and does not stain non-sensory supporting cells within the neuromast. This is due to supporting cells lacking similar mitochondrial density and electrochemical potential profile as the sensory hair cells, preventing DASPEI accumulation [20,21]. As a result, supporting cells cannot be counted using DASPEI or other mitochondrial-dependent dyes. During the analysis of neuromast cell regeneration and development, neomycin exposure resulted in rapid hair cell loss as early as 4 h post-treatment, with the most pronounced effect observed at 24 h, where neuromast cells were completely absent (Figure 2B and Figure 4B). While the data remain qualitative regarding regenerative capacity, neuromast cells were more consistently observed in the infraorbital bone3 region compared to the body, suggesting spatial variation in neuromast regeneration. This pattern implies that not all neuromast cells regenerate at the same rate (Figure 2 and Figure 4). The absence of detectable signal in some regions may reflect limited hair cell regeneration over the 72 h period, as DASPEI is a live stain specific to sensory hair cells, and reduced hair cell numbers can result in diminished signal detection.

Our findings, together with previous reports, confirm that Mexican tetra surface fish neuromast hair cells are acutely sensitive to aminoglycoside exposure and are able to rapidly regenerate in as little as 72 h post-exposure. Neomycin exposure produces a characteristic pattern of hair cell death within the first few hours, consistent with mitochondrial-driven cytotoxicity. Specifically, neomycin induces endoplasmic reticulum (ER) mitochondrial calcium transfer through IP3 receptors, leading to mitochondrial calcium overload, loss of mitochondrial membrane potential, and accumulation of reactive oxygen species (ROS), ultimately causing rapid mitochondrial collapse and hair cell death [22,23]. Neuromast hair cells are not permanent. Similarly to our model in zebrafish, the neomycin exposure eliminates most lateral line hair cells within 1–4 h, but supporting cells re-enter the cell cycle within 12–24 h post-injury [24]. By 24–48 h, new hair cells emerge, demonstrating differentiation from supporting cells into functional mechanoreceptors [25,26]. By 72 h post-exposure, neuromasts typically contain hair cell numbers comparable to untreated controls, and functional recovery of mechanosensory responses has been demonstrated at this stage [27]. Thus, the regenerative process progresses from acute cell death to full replacement within approximately three days.

The mechanisms underlying this regenerative capacity rely heavily on supporting cells acting as progenitors. Evidence indicates that regeneration can occur via two parallel routes: (1) direct transdifferentiation of supporting cells into hair cells, and (2) mitotic proliferation of a subset of supporting cells, followed by differentiation [28,29]. These processes are tightly regulated by signaling pathways. Transcription factor *Atoh1* activates cell proliferation and promotes hair cell fate in the mammalian cochlear epithelium [30]. The balance between these pathways is critical to both the pace and fidelity of regeneration. Comparatively, the ability of zebrafish to regenerate neuromast hair cells within 72 h contrasts sharply with mammalian systems, where sensory hair cell loss is permanent due to the absence of regenerative competence in supporting cells after early development [6]. The differences suggest that while signaling pathways are conserved, their regenerative potential has been silenced in mammals. Therefore, while a model for regenerative research does exist through zebrafish, the Mexican tetra as a future model provides an essential framework for dissecting the cellular and molecular determinants of regeneration through an evolutionary perspective. Comparing two closely related organisms that have diverged in separate environments to adapt to one’s surroundings, with cavefish heavily relying on sensory adaptations versus surface fish [31,32].

Together, these results highlight that neuromast regeneration is not only rapid but also highly coordinated, with a defined temporal sequence beginning within 24 h of injury and reaching completion by 72 h after the injury, which highlights the robustness of the regenerative potential of the Mexican tetra neuromast cell structure. This further accounts for the importance of these structures for the continuous survival of the animal in the aquatic environment. This regenerative timeline should be considered in the design of ototoxicity assays and therapeutic interventions, as it defines the window for observing both acute damage and subsequent repair.

When looking at therapeutic interventions for acute damage and repair, it is important to understand the underlying pathways and their potential involvement within the regenerative process. Therefore, Wnt signaling was looked at through *axin2* expression, as well as *fgf1* due to their important significance in activation of the neuromast cell regenerative process [33,34,35,36]. Wnt signaling is mediated through the downstream activation of *axin2*, playing a central role in stimulating progenitor cell proliferation. Activation of Wnt/β-catenin signaling rapidly induces expression of *axin2*, which serves as both a marker of Wnt pathway activity and a negative feedback regulator, ensuring that proliferative responses remain controlled [36]. This proliferative burst is tightly coupled with Fgf signaling, as Wnt activity alone is insufficient to sustain regeneration without Fgf pathway involvement. Indeed, Wnt activation upregulates *fgfr1*, and inhibition of Fgf disrupts both proliferation and hair cell replacement, underscoring the synergistic relationship between these two pathways [35,36]. Thus, Wnt–Fgf crosstalk defines an early regenerative axis, coordinating progenitor expansion and subsequent differentiation.

While *sox2* has not been directly shown to be involved in neuromast cell regeneration, previous studies have demonstrated its importance within supportive cells surrounding the neuromast for the regenerative process [25,35,36,37,38]. Therefore, changes in *sox2* expression were analyzed in this study on surface fish. *sox2* expression marks a supporting cell population that serves as the progenitor pool for hair cell regeneration. *sox2* supporting cells are maintained in a poised state, capable of either directly transdifferentiating into hair cells or undergoing proliferation before differentiation [29]. *sox2* contributes to the regulation of Wnt and Fgf activity, acting as a molecular integrator that balances progenitor identity with regenerative capacity [29,36].

Wnt activation was witnessed in Figure 6 when analyzing the gene expression changes associated with *axin2*, with *fgf1* expression also increasing 72 h post-neomycin-treatment. Although one-way ANOVA followed by Tukey’s post hoc test did not reveal statistically significant differences among groups (*p* > 0.05), observable trends in gene expression suggest potential biological relevance. fgf1 expression demonstrated a time-dependent increase, rising from mean expression level of 0.07 at 24 h to 0.1 at 72 h post-treatment (Figure 6). This progressive upregulation may indicate a role for fgf1 in promoting regenerative signaling, consistent with its known involvement in cell proliferation and tissue repair. Similarly, *axin2* expression showed a modest elevation at 24 h, suggesting early activation of Wnt signaling pathways, which are often implicated in stem cell regulation and regeneration. In contrast, *sox2* expression remained relatively stable across all time points. Given the role of *sox2* in maintaining stem cell pluripotency, its consistent expression may reflect a steady-state requirement for neuromast progenitor maintenance rather than dynamic regulation in response to injury. These findings underscore the need for further investigation into the molecular mechanisms driving neuromast regeneration, including the potential involvement of alternative signaling pathways or post-transcriptional regulatory processes that may not be captured through gene expression analysis alone. Increasing sample size or incorporating additional time points may help clarify the roles of these genes in the regenerative process. Moreover, complementary approaches such as protein-level analysis or pathway-specific inhibition could provide deeper insights into the functional relevance of these transcriptional changes.

## 5. Conclusions

In conclusion, this study highlights the use of Mexican tetra as a model organism to study the development and regeneration of the sensory hair cells and provides a comprehensive view of hair cell vulnerability and recovery following neomycin-induced damage. The use of DASPEI staining enabled precise temporal assessment of neuromast viability across multiple post-exposure intervals, revealing dynamic patterns of degeneration and potential regeneration. The modest upregulation of *axin2* and *fgf1* gene expression, alongside the stable levels of *sox2* across all time points following neomycin treatment, suggests limited transcriptional response within neuromast regeneration. This study demonstrates the value of *Astyanax mexicanus* as a model for investigating ototoxicity and sensory hair cell regeneration.

## Figures and Tables

**Table 1:** Specific primers details are presented in Table 1.

*fgf1a* (XM_007258578.4)	Forward primer	GGCACGAGACCGGACGTTTC
	Reverse primer	GCCGTTCTTTGTCTGCCCAC
*axin2* (XR_007427266.1)	Forward primer	GCGCGGATCGATGGTAAATA
	Reverse primer	CCCTGTTCATGGCTCGGG
*sox2* (NM_001319965.1)	Forward primer	TCATCGGCTCTTCGGAGGTTT
	Reverse primer	ACATCCTCCCATGCACCTGTT

## Data Availability

The original contributions presented in this study are included in the article/Supplementary Material. Further inquiries can be directed to the corresponding author.

## Supplementary Materials

The following supporting information can be downloaded at: https://www.mdpi.com/article/10.3390/cells14211680/s1, Figure S1: Whole-mount live cell staining of surface fish using Alizarin Red and DASPEI to visualize superficial and bony canal neuromast cells. Alizarin Red marks mineralized craniofacial structures, while DASPEI labels metabolically active neuromast cells, enabling clear identification of both superficial and canal-associated sensory organs. (A) Craniofacial bone staining with Alizarin Red. (B) Neuromast cell staining with DASPEI under GFP filter. (C) Merged image of both channels, revealing cranial neuromast cells within bony canals and superficial neuromasts along the infraorbital bones and facial surface. Infra orbital bone3 (IO3), Neuromast cells (NM) Scale bar: 1000 µm; Figure S2: Confocal imaging of neuromast cell structure in surface fish following live DASPEI staining. The spatial organization of neuromasts within infraorbital bone3 is visualized, including the superficial neuromast (NM).
