# Supplementary material for: Spatial Distribution and Temporal Dynamics of Neomycin-Induced Neuromast Cell Damage and Regeneration in the Mexican tetra (Astyanax mexicanus)
Source: Cells. 2025 Oct 27;14(21):1680. doi: 10.3390/cells14211680 (PMC12607775; doi:10.3390/cells14211680)
Supplement: Supplementary file 1 [file cells-14-01680-s001.zip › cells-3926121-supplementary.pdf]

Supplementary File

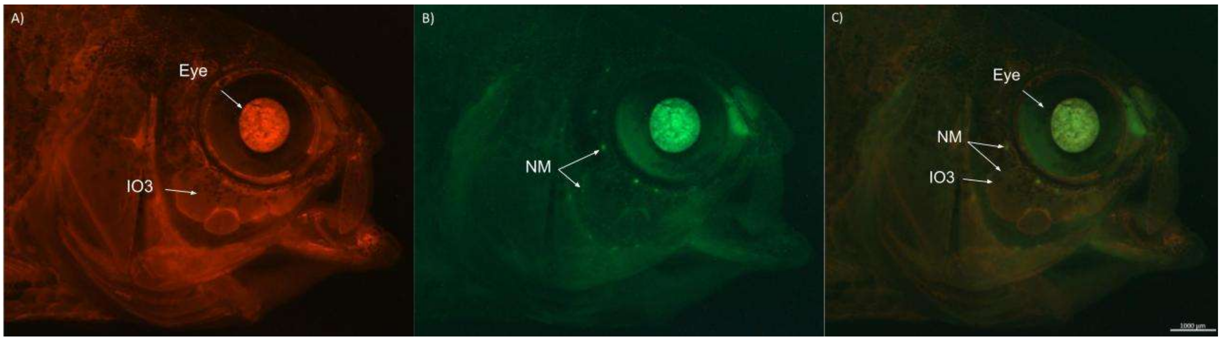

Figure S1. Whole-mount live cell staining of surface fish using Alizarin Red and DASPEI to visualize superficial and bony canal neuromast cells. Alizarin Red marks mineralized craniofacial structures, while DASPEI labels metabolically active neuromast cells, enabling clear identification of both superficial and canal-associated sensory organs. A) Craniofacial bone staining with Alizarin Red. B) Neuromast cell staining with DASPEI under GFP filter. C) Merged image of both channels, revealing cranial neuromast cells within bony canals and superficial neuromasts along the infraorbital bones and facial surface. Infra orbital b one 3 (IO3), neuromast cells (NM). Scale bar: 1000 µm.

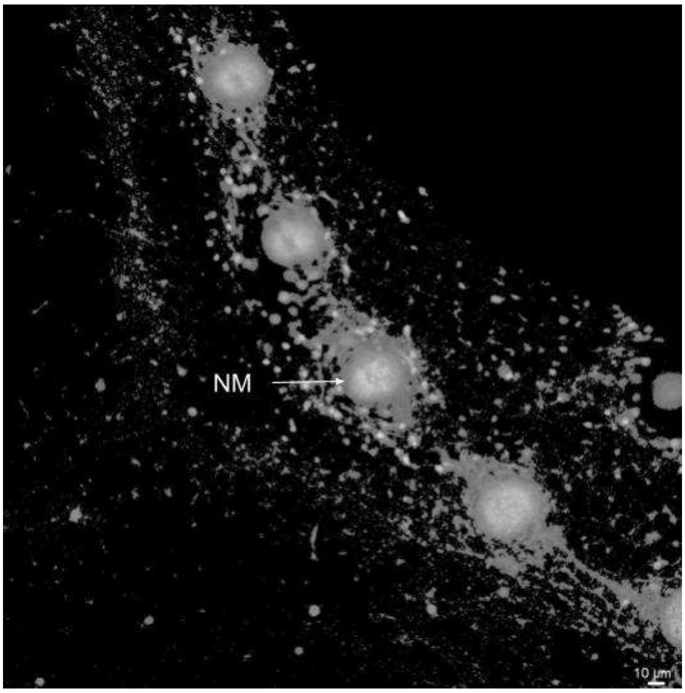

Figure S2. Confocal imaging of neuromast cell structure in surface fish following live DASPEI staining. The spatial organization of neuromasts within infraorbital bone 3 is visualized, including the superficial neuromast (NM).
